# Qualitative Evaluation of the Personal KinetiGraph^TM^ Movement Recording System in a Parkinson’s Clinic

**DOI:** 10.3233/JPD-181373

**Published:** 2019-02-05

**Authors:** Anthony Santiago, James W. Langston, Rita Gandhy, Rohit Dhall, Salima Brillman, Linda Rees, Carrolee Barlow

**Affiliations:** a Parkinson’s Institute and Clinical Center, Sunnyvale, CA, USA; b Formerly of Parkinson’s Institute and Clinical Center, Sunnyvale, CA, USA; c Department of Neurology, University of Arkansas, Little Rock, AR, USA

**Keywords:** Parkinson’s disease, remote monitoring, wearable sensors, objective assessment, continuous measurement, capacitive sensor

## Abstract

**Background:**

Wearable sensors provide accurate, continuous objective measurements, quantifying the variable motor states of patients with Parkinson’s disease (PD) in real time.

**Objectives:**

To evaluate the impact of using continuous objective measurement using the Personal KinetiGraph™ (PKG^®^) Movement Recording System in the routine clinical care of patients with PD (PwP).

**Methods:**

Physicians employed the use of the PKG in patients for whom they were seeking objective measurement. Patients wore a PKG data logger for ≥6 days during routine daily living activities. During the survey period of December 2015 through July 2016, physician surveys were completed by four Movement Disorder Specialists for whom measurements from the PKG were available during a subsequent routine clinic visit.

**Results:**

Of 112 completed physician surveys, 46 (41%) indicated the PKG provided relevant additional information sufficient to consider adjusting their therapeutic management plan; 66 (59%) indicated the PKG provided no further information to support a therapeutic decision differing from that made during a routine clinical evaluation. Upon further review of these 46 surveys, 36 surveys (78%) revealed the information provided by the PKG ultimately resulted in adjusting the patient’s medical management.

**Conclusions:**

The PKG provided novel additional information beyond that captured during a routine clinic visit sufficient to change the medical management of PwP. Physicians adjusted treatment nearly a third of the time based on data provided by real-time, remote monitoring outside the clinic setting. The use of the PKG may provide for better informed therapeutic decisions, improving the quality of life for PwP.

## INTRODUCTION

Parkinson’s disease (PD) is a chronic, progressive, neurodegenerative movement disorder characterized, in part, by motor symptoms of bradykinesia, muscular rigidity, and tremor. Clinical manifestation and course of disease progression varies considerably across PD patients.

While care of patients with Parkinson’s disease (PwP) is highly individualized and requires consideration of a variety of motor and non-motor symptomatology (sensory, neuropsychiatric, autonomic, etc.), pharmacologic therapeutic intervention aimed at motor symptom control is typically a key part of disease management. While clinicians rely on clinical observation during a clinic visit, typically the patient’s interim history and recall are heavily weighted when making therapeutic decisions such as medication adjustments. However, when it comes to patient reporting, motor symptom recall can be incomplete and inaccurate (e.g., mistaking dyskinesia for tremor, or being unaware of dyskinesia), and symptoms and response to therapy, which may vary in frequency and severity from day to day are often difficult to recall in any detail. As noted above, a typical clinical visit provides only a snapshot of these signs, symptoms, and variations in response to therapy over time.

Incomplete patient reporting is not a trivial issue, as an accurate evaluation of the PD disease state and response to medication over time is critical for improving diagnosis and staging, monitoring response to therapy and motor complications, improving medical treatment, enhancing surgical treatment decisions and improving rehabilitation interventions [1, 2]. More importantly, discrepancies in reporting any of the features in the day-to-day management of PD may lead to the wrong changes in medications.

New wearable sensors have the advantage of offering continuous objective measurement of patient movement during regular activities of daily living, and therefore, have the potential to provide important additional information in a more accurate way to augment clinical care of PD patients. Over the past two decades, wearable technologies have become of increasing interest due to the lack of measurement tools to objectively quantify PD motor symptoms over much longer times than the brief window of time spent in a doctor’s office [3–5]. More importantly, recent studies suggest the use of wearable sensors can inform medication choices and correlate with relevant clinical assessment scales [6, 7]. Wearable sensors have also been shown to be well tolerated by patients and the availability of personalized information can enhance patient quality of life [8]. Some of these technologies are now making the transition from research into routine clinical care.

One of these technologies is the FDA-cleared wearable Personal KinetiGraph^™^ Recording System (PKG^®^) Movement Recording System (PKG System), which is intended to quantify kinematics of movement disorder symptoms in conditions such as PD, including tremor, bradykinesia and dyskinesia; it includes a medication reminder, an event marker, and is intended to monitor activity associated with movement during sleep. The PKG System is indicated for use in individuals 46 to 83 years of age and was FDA cleared on August 22, 2014 (K140086). The PKG System consists of an interactive data logger (PKG Watch) that resembles a wristwatch and is worn on the right or left wrist of the body side most severely impacted by PD. It contains a battery, accelerometer, memory, an optional reminder to the subject when PD medications are due and a means for recording when PD medications are taken, as well as a capacitive sensor that detects removal of the watch from the wrist. Clinic staff configure a PKG data logger for each patient prior to its use with a simple-to-use program on a tablet computer. During patient wear time of six or more days, the PKG data logger continually collects data on patient movement and reminds the patient to acknowledge when to take their levodopa medication.

At the end of the patient wear period, the PKG data logger is returned to the clinic and data are downloaded and analyzed using the manufacturer’s (GKC) algorithm to translate raw movement data into a printable output (PKG) of the patient’s movement over the wear period. The PKG includes daily and summary scores for bradykinesia (BKS), dyskinesia (DKS), and fluctuations (FDS), data on tremor, immobility, movement during daytime, somnolence, sleep, and a summary of any periods in which the PKG data logger was off-wrist. The resulting PKG is then reviewed by a Movement Disorder Specialist (MDS) and the information used along with information gained during the routine visit to make clinical decisions.

Several published validation studies demonstrate the PKG System’s ability to quantify the kinematics of movement disorder symptoms in PwP. A validation study published in 2012 described methodology behind the PKG bradykinesia and dyskinesia algorithms and compared these outcomes with existing clinical scales. The PKG BKS was shown to closely correlate with the Unified Parkinson’s Rating Scale Part III (UPDRS III) motor score (minus tremor item) and the PKG DKS correlated with the modified Abnormal Involuntary Movement Score (AIMS) [9]. Evans, et al. demonstrated overuse of the medication acknowledgement on the PKG Watch converted to a response ratio (number of acknowledgements/number of doses) correlated in 19 of 25 subjects to impulsive-compulsive behavior (ICB) scales, suggesting an elevated PKG response ratio may be an indication of ICBs, which are often underreported in PwP [10]. The PKGs FDS was validated by Horne, et al. who describe a highly sensitive (97.1%) and selective (87.5%) score derived from BKS and DKS scores for objectively identifying PD-related fluctuations. The authors conclude the FDS promises to be a useful tool for identifying patients whose fluctuations are progressing and may require therapeutic changes [11]. Ossig, et al. compared PKG motor assessments with patient-completed home diaries. Distribution of total hours per day in all motor states measured by PKG closely reflected those assessed by PD home diaries. Consistently, the authors found a moderate correlation between calibrated PKG and diary data for total daily hours in Off and On state without dyskinesia and a strong correlation for the dyskinetic state on the group level. Authors conclude the PKG is a valuable tool to measure total motor state hours per day particularly for dyskinesias [12]. A series of published studies provided further validation of PKG use in the routine care of PwP. PKG measures of bradykinesia and dyskinesia were shown to correlate with relevant clinical scales and capture the effect of therapeutic interventions [6]. Price, et al. evaluated the clinical utility of the PKG in routine clinical care and found the PKG assisted in patient education, explaining treatment plans, and facilitating discussion around symptom management. Perhaps most importantly, in 63% of patients, the PKG identified issues that had not been reported previously [13]. Spengler, et al. reported PKG use to support deep brain stimulation (DBS) programming was feasible and may decrease time-to-DBS optimization contributing to a more effective DBS therapy and possibly fewer programming visits [14]. Additionally, a recent study using the PKG showed how a cohort of PwP with uncontrolled PD symptoms would benefit from objective assessment treatment of their PD features towards a target range [15].

The Parkinson’s Institute and Clinical Center began using the PKG System in December 2015 as an adjunct to the clinical visit history and examination. In this qualitative evaluation, we assessed the “real world” ways in which the PKG could support clinical management decisions in day-to-day patient care by physicians who were using the PKG to assess impact of this new tool on clinical decisions and patient care.

## MATERIALS AND METHODS

### Study objective

The main objective of this study was to evaluate the impact of using continuous objective measurement provided by the PKG in the routine clinical care of PwP.

### Study design

This was a prospective physician survey study. Four MDSs at our clinic previously completed training on the use and reporting of the PKG, and began using the PKG in the routine clinic setting for PwP. These MDSs had varying levels of overall clinical experience and experience using the PKG. MDSs were asked to complete a survey after each patient visit in which the PKG was used in routine care. No additional clinical or research tools or assessments were used.

### Patient selection

Because these patients were already being seen in the clinic under typical clinic monitoring for PD patients, the project was exempt from Institutional Review Board approval and patient informed consent.

In routine care, physicians targeted PKG use in patient populations they believed continuous objective measurement would improve the value of clinical encounters. Patients generally fell into four categories as listed below.

1. **First Patient Visit in Clinic**: Patients were new to the clinic, that is, the first time seen in clinic. Continuous objective measurement data on the first visit could potentially make it easier to determine what types of medication changes or next steps in medical management were needed (given physicians had no prior history with the patient).
2. Patients with **PD Symptom Fluctuations** related to therapy: Patients experiencing clear wearing off, prolonged offs, or dyskinesia, or recent changes in PD symptom response to medication. Continuous objective measurement data may assist with system discovery in patients who are not aware fluctuations are occurring.
3. Patients with **Indeterminate History**: Patients who were unable to clearly articulate type, severity, frequency, and/or duration of their PD symptoms in response to medication. Continuous objective measurement data may assist with objective quantification and summary of PD symptoms that could be used during the clinical encounter with the patient to facilitate discussion and support clinical decision making.
4. Patients considering or using **Deep Brain Stimulation (DBS) or Duopa**: Patients in contemplation of or being evaluated for DBS or Duopa, or patients who are already using these therapies to assess the potential need for adjustments. Continuous objective measurement may provide an additional level of confidence for advanced therapy selection by illustrating and quantifying dose-response and response duration.

### The Physician PKG Survey

The Physician PKG Survey consisted of a reason for the clinical encounter, existing PD medications at the time of the encounter for any patients new to the clinic and clinical management changes made following the routine follow-up evaluation. Upon review of the PKG data by the physician, an assessment was then made on whether the PKG provided, or did not provide, information not available from the clinical consultation alone, and if that information played a role in making decisions on clinical management moving forward. The two main questions asked on the physician survey were:

1. “Did the PKG provide additional information” – Information provided by the PKG was compared and contrasted to physician observation and patient-reported history.
2. “Was a clinical management plan changed made”- Examples of such decisions included changes in PD medication type, dose, frequency, amount per dose, or referral for advanced therapies (e.g., DBS, Duopa).

Patients often wore the PKG the preceding two to three weeks before a routine clinic visit and therefore the physician had the PKG data available for review at the clinic visit, which was the target work flow. In cases where the patient did not wear the PKG prior to a routine clinic visit, the patient was provided with a PKG data logger immediately following a clinic visit. In these cases, the physician would complete the survey after any routine post-visit follow-up with the patient was complete and the PKG had been worn for at least six days after consultation. The physician would at that time review the PKG data and complete the survey.

### Analysis of data

Relevant summary statistics were reported.

## RESULTS

This project was conducted under the supervision of Parkinson’s Institute and Clinical Center’s clinical and administrative leadership. The extent of the four-physician participation varied from 6 surveys to 46 surveys per physician among the final set of completed surveys.

During the survey period, December 2015 through July 2016, a total of 143 PKGs were ordered on 89 patients (44 patients had two or three PKGs) as part of their routine clinical evaluation and follow-up PD care visits. Of the 89 patients, 45 had one PKG, 44 had two PKGs, and 10 of the 44 patients went on to have three PKGs completed. A total of 119 Physician Surveys were completed. Four additional Physician Surveys were partially completed (having answered one of the two key questions) and for 20 patient visits where a PKG was used, a physician inadvertently did not complete the survey. Therefore, the survey completion rate was 83% (119/143). Survey completion rates were the highest for patients with only one PKG (97%) and decreased with the 2nd PKG to 64% and then to 50% at the 3rd PKG. Table 1 *(Number of PKGs and Physician PKG Surveys Completed)* summarizes PKG and Physician PKG Survey completion numbers by patient sub-group.

**Table 1:** Number of PKGs and Physician PKG Surveys Completed

Patient Group	Sequence Number	Number PKGs Completed	Number Fully Completed Surveys
First Patient Visit in Clinic	1st	39	38
	2nd	23	14
	3rd	6	4
	Group Total	68	56
PD Symptom Fluctuator Patients	1st	39	37
	2nd	17	12
	3rd	3	1
	Group Total	59	50
Indeterminate History Patients	1st	8	8
	2nd	3	1
	3rd	1	0
	Group Total	12	9
DBS/Duopa Patients	1st	3	3
	2nd	1	1
	3rd	0	0
	Group Total	4	4
All Patients	1st	89	86
	2nd	44	28
	3rd	10	5
	Overall Total	143	119

Of the 119-completed physician PKG surveys, seven were removed from analysis because they were completed prior to the first time in clinic visit, leaving a total of 112 completed surveys in 81 patients to be included in the final analysis.

Of these 112 physician surveys, 46 (41%) indicated the PKG provided additional information to the physician; however, 66 (59%) indicated the PKG provided no additional information. Upon further review of the 46 surveys, 36 surveys [36/46 (78%)] stated the information resulted in an alteration in patient care whereas, 10 surveys [10/46 (22%)] stated the PKG provided additional information but that no alteration in patient care occurred based on this information. For the overall cohort, 32% (36/112) resulted in alteration to patient care and 9% (10/112) no alteration to patient care.

Of the 36 patients the PKG most commonly yielded new and precise information on daily off time [50% of cases (18/36)]. Table 2 is a summary of the Physician PKG survey results by patient group and PKG sequence. When all patients are considered as a group, both the first and second PKG resulted in a 33% alteration in patient care.

**Table 2:** Summary of Physician PKG Surveys by Patient Group and Sequence Number

Patient Group	PKGs Sequence	Completed Surveys (N)	Physician PKG Surveys				Percent Alteration in Patient Care by Sequence and Patient Group
			Did the PKG Provide Additional Information? (N)		Was a Clinical Management Plan Change Made? (N)		
			Yes	No	Yes	No
First Patient Visit in Clinic	1st	33	8	25	4	4	4/33 (12%)
	2nd	13	5	8	4	1	4/13 (31%)
	3rd	3	0	3	0	0	–
	Group Total	49	13	36	8	5	8/49 (16%)
PD Symptom Fluctuator Patients	1st	37	19	18	17	2	17/37 (46%)
	2nd	12	5	7	4	1	4/12 (33%)
	3rd	1	0	1	0	0	–
	Group Total	50	24	26	21	3	21/50 (42%)
Indeterminate History Patients	1st	8	6	2	4	2	4/8 (50%)
	2nd	1	1	0	1	0	1/1 (100%)
	3rd	0	0	0	0	0	–
	Group Total	9	7	2	5	2	5/9 (56%)
DBS/Duopa Patients	1st	3	2	1	2	0	2/3 (67%)
	2nd	1	0	1	0	0	–
	3rd	0	0	0	0	0	–
	Group Total	4	2	2	2	1	2/4 (50%)
All Patients	1st	81	35	46	27	8	27/81 (33%)
	2nd	27	11	16	9	2	9/27 (33%)
	3rd	4	0	4	0	0	–
	Overall Total	112	46/112 (41%)	66/112 (59%)	36/112 (32%)	10/112 (9%)	36/112 (32%)

Of the 36 total surveys reporting an alteration in patient care, the PD Symptom Fluctuator patient group had the largest proportion of surveys at 58% (21/36) followed by the First Patient Visit in Clinic patient group at 22% (8/36), the Indeterminate History patient group at 14% (5/36), and DBS/Duopa patient group at 6% (2/36).

In the PD Symptom Fluctuator patient group, changes to PD medications were the reason for all alterations in patient care. In the next section, we present three case study examples from patients in the Fluctuator patient group depicting PKG information which informed patient care. The first case demonstrates management of peak-dose dyskinesia; the second case highlights managing wearing-off without worsening existing dyskinesia; and the third case shows the patient’s subjective report of markedly wearing-off and disabling bradykinesia, is revealed as predominantly marked dyskinesia requiring further management.

### Case studies

#### Case 1 Patient No 18

**Fig. 1:**
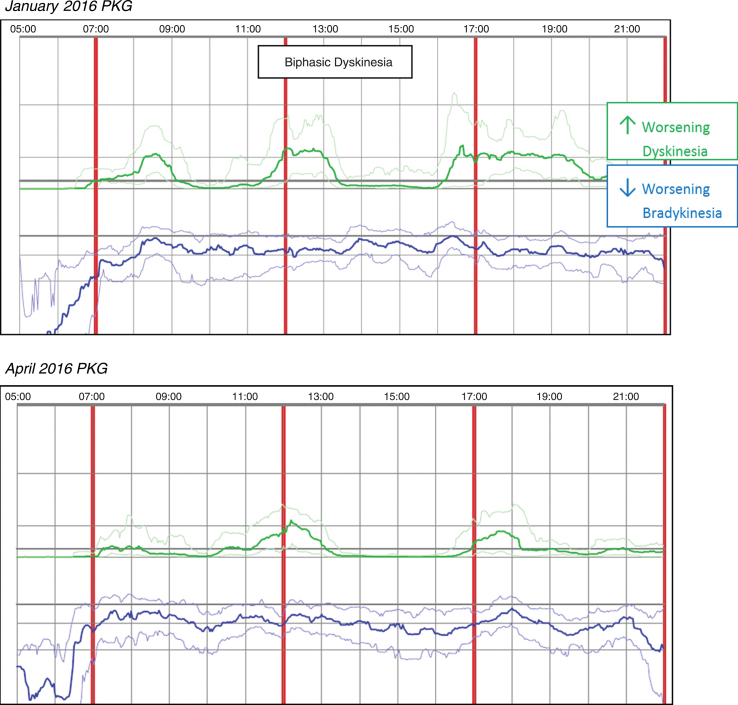
Patient No 18 January 2016 and April 2016 PKG. PKG Summary Plot depicts data from recording day aligned to the time of day. It shows when reminders were given (vertical red lines), the median DKS (heavy green line) and median BKS (heavy blue line) and their corresponding 25th and 75th percentiles plotted against time of day.

Case 1 was taking Rytary 95 mg, two capsules QID and a monoamine oxidase inhibitor (MAOI). The patient reported mild wearing-off and dyskinesia, along with gait challenges and leg dystonia. The initial PKG in January 2016 revealed symptoms more severe than reported with DKS that peaked about 90 minutes after the first dose which usually fell to near normal levels within 30–60 minutes and biphasic dyskinesia was also noted. Median DKS was 4.0 and the FDS was 11.8 indicating fluctuations were present. An anti-dyskinetic (propranolol 10 mg Qam) was added to the medication regimen during the January office visit; in March of 2016, Amantadine 100mg BID was added based on the patient phone report of continued dyskinesia. A subsequent April 2016 PKG, performed days prior to the office visit, revealed median DKS was reduced to 1.6 and FDS to 8.6. Median BKS increased slightly from 23.3 in January, to 25.7 in April; however, dyskinesia fluctuations are less pronounced. Dose-related dyskinesia is still present but reduced in severity and duration, consistent with the patient report of doing better. Figure 1 *(Patient No 18 January 2016 and April 2016 PKG)* depicts the two PKG printouts.

#### Case 2 Patient No 13

**Fig. 2:**
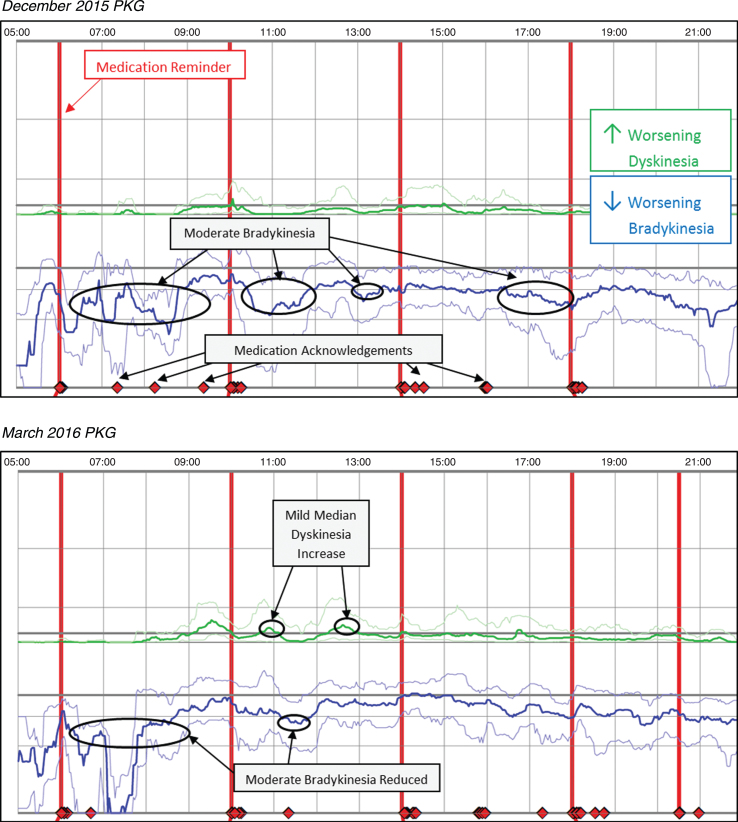
Patient No 13 December 2015 and March 2016 PKG. PKG Summary Plot depicts data from recording day aligned to the time of day. It shows when reminders were given (vertical red lines), the median DKS (heavy green line) and median BKS (heavy blue line) and their corresponding 25th and 75th percentiles plotted against time of day.

Case 2 was prescribed Sinemet 25/100 one tablet five times daily, a MAOI (rasagiline 1mg), and Sinemet 50/200 CR at bedtime; the patient reported PD medication was not lasting, with greater time feeling off. Upon further questioning, it was revealed the patient had not been taking prescribed medication consistently. The initial PKG in December 2015 revealed a median BKS of 26.4 and no or limited dose response, and fluctuating symptoms with periods of moderate bradykinesia. The PKG also indicated the patient frequently acknowledged taking medication outside the prescribed dose regimen. Based on the patient report and the initial PKG, the patient was educated to the importance of consistently taking medication as prescribed. At the March 2016 visit the subsequent PKG showed improved medication compliance and the median BKS reduced to 22.5 without a substantial increase in dyskinesia. Overall, the patient was spending ∼51% of the waking day in moderate-to-severe levels of bradykinesia in December 2015, which was reduced to ∼37% in March 2016 (vs ∼25% for control patients). Figure 2 (*Patient No. 13 December 2015 and March 2016 PKG*) depicts the two PKG printouts.

#### Case 3 Patient No 145

**Fig. 3:**
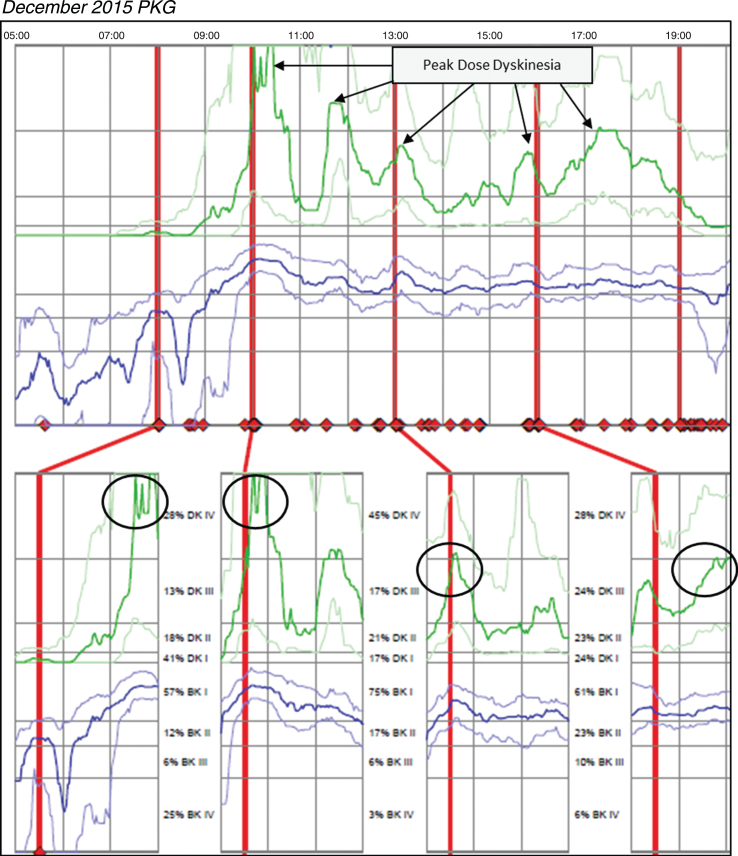
Patient 145 December 2015 PKG: PKG Summary Plot depicts peak dose dyskinesia. Top Image - PKG Summary Plot: Depicts data from recording day aligned to the time of day. It shows when medication reminders were given (vertical red lines), the median DKS (heavy green line) and median BKS (heavy blue line) and their corresponding 25th and 75th percentiles plotted against time of day. Bottom Image - PKG Dose Response Curves: Depicts data from recording day aligned to the time of medication acknowledgement for each individual dose. Programmed medication doses are depicted by vertical red lines. Also illustrated are the median DKS (heavy green line) and median BKS (heavy blue line) and their corresponding 25th and 75th percentiles plotted against time of day.

**Fig. 4:**
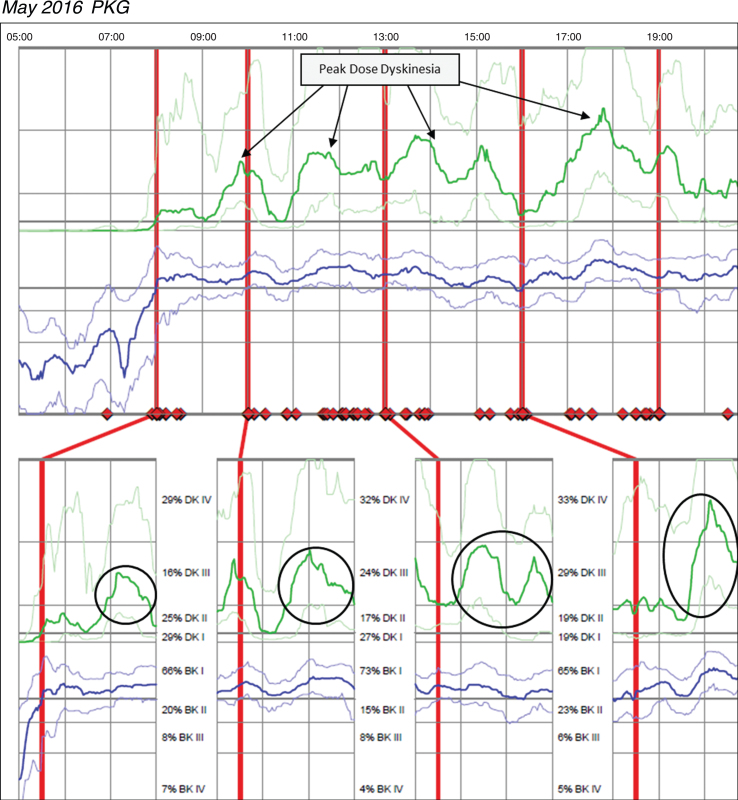
Patient 145 May 2016 PKG. PKG Summary Plot depicts peak dose dyskinesia. Top Image - PKG Summary Plot: Depicts data from all recording day aligned to the time of day. It shows when reminders were given (vertical red lines), the median DKS (heavy green line) and median BKS (heavy blue line) and their corresponding 25th and 75th percentiles plotted against time of day. Bottom Image - PKG Dose Response Curves: Depicts data from recording day aligned to the time of medication acknowledgement for each individual dose. Programmed medication doses are depicted by vertical red lines. Also illustrated are the median DKS (heavy green line) and median BKS (heavy blue line) and their corresponding 25th and 75th percentiles plotted against time of day.

Case 3 was taking Sinemet 25/100 two tablets five times per day and Sinemet 50/200 CR ½ tablet TID and one tablet QHS. The patient reported significant motor fluctuations with wearing-off, delayed-on and challenges performing activities of daily living relative to bradykinesia. The patient was unable to say with certainty how long doses of medication last, but the patient did not report challenges with dyskinesia. Based on patient’s report, no change was made in medication and a PKG was ordered after this December 2015 office visit. Figure 3 (*Patient 145 December 2015 PKG*) depicts dyskinesia even though the patient did not report it. The PKG revealed a BKS score of 14.8 which is below the median for controls and approximately 68% of the day in BKI (vs ∼50% for controls), and frequent and significant fluctuations with markedly elevated median DKS of 24.8 and FDS of 19.2. The patient was contacted by phone and instructed to reduce his Sinemet 25/100 IR to one tablet five times per day and increase Sinemet 50/200 CR ½ tablet from three times to five times per day coinciding with the Sinemet IR dosing. The patient subsequently reported by phone his delayed-on and dyskinesia were both worse and was instructed by phone to return to his original medication regimen. This resulted in worsening dyskinesia and a subsequent PKG was ordered in May 2016; during his June 2016 visit, the results were reviewed and the options of DBS and Duopa were discussed to treat his significant motor fluctuations. Figure 4 *(Patient 145 May 2016 PKG)* depicts dyskinesia from the May 2016 PKG.

## DISCUSSION

In this qualitative evaluation, Movement Disorder Specialists in a Parkinson’s disease clinic reported the PKG provided additional information beyond that obtained during clinical consultation alone in 41% of visits, and resulted in adjusting treatment nearly a third of the time overall. The PKG most commonly yielded new and precise information on daily off time [50% of cases (18/36)], which suggests a key area where clinical consultations can derive value from the addition of continuous objective measurement.

Use of the PKG in new patient exams were found to be more beneficial when the PKG was given to the patient after the first new patient visit. Given the comprehensive data collection and information already covered at the first new patient visit, the participating physicians felt more comfortable using methods already established for the first new patient consults.

This work represents the first systematic evaluation of physician assessment on the value added by continuous objective measurement to the clinical consultation in a “real world” clinical practice in the United States. As such, these data offer new insight into the role of such technologies in the routine care of patients with PD. While many segments of PD research focus on the development of new therapies, we have learned there is also an opportunity to maximize the use of existing PD therapies in routine clinical care. Continuous objective measurement may offer a mechanism to objectively define PD motor symptoms, define treatment targets for motor function, and lead to a new paradigm for better monitoring PD patients, which may ultimately drive improved use of treatments we already have in our armamentarium as well as better outcomes for PD patients.

Integration of continuous objective measurement technology into routine clinical care required initial support staff training from the device manufacturer related to device logistics (dispensing, wear, retrieval) and physician training on clinical interpretation of the device data summary provided at the end of the wear period. Physicians also benefited from a deeper dive into the science behind how the technology works and the summary data are generally derived. Physicians completed an initial evaluation phase with report interpretation support and the learning curve for each physician varied. Clinical work flow development was required to define how the device would be ordered and provided to the patient with the aim of having the device data available at the time of the clinic visit with the patient as we found in many instances that reviewing the PKG data with the patient during the clinic visit assisted with symptom discovery. Patient training was completed in the clinic or remotely depending on the patient’s preference and familiarity with the device.

### Limitations

Some caveats deserve mention. This was a qualitative physician evaluation aimed at assessing clinical utility of the PKG in routine clinical care, and as such, detailed data collection and research-driven subject visit schedules were not employed, which limits the ability to expound further on the results. Level of physician engagement in the project varied. Physician assessment of clinical value derived from continuous objective measurement use may have been limited by the extent of physician familiarity and knowledge of product use and interpretation (per physician use ranged from 6 to 46 PKGs during the evaluation period), variation in duration between the clinic visit and survey completion, and logistical complexity of adding new technology into existing clinical practice flow. This study was the participating physician’s early experience using the PKG Movement Recording System. Serial PKGs were not completed on all patients but it is suspected there would be a learning curve regarding proficiency and data interpretation with the PKG device. Given the taper of serial PKGs, this was difficult to assess. Also, while the serial PKG results are informative, we are limited in our ability to draw substantial conclusions regarding a clinical care construct for using serial PKGs in routine clinical care.

### Generalizability

While results from this project are the clinical impressions of four physicians from one institution, the project reflects real-life in that it includes typically-encountered PD patients along the continuum of disease, and physicians with varying levels of clinical experience with continuous objective measurement. Data collected in this project suggests wearable technology, like the PKG, has a role to play in routine care of PD patients at tertiary care centers such as movement disorders clinics, and may change the way we monitor motor symptoms in PD patients. Continuous objective measurement has the potential to add value in non-specialty care centers as well, where a substantial portion of the PD patient population receives care and providers generally see a smaller overall percentage of PD patients within their population. Therefore, continuous objective measurement may provide even greater support to physicians and patients for PD symptom identification, patient counseling, and treatment planning.

### Future directions

Clinical management of PD will continue to involve qualitative and quantitative assessments, but the challenge is the inability to gather relevant and accurate clinical information that reflects the majority of the time the patient is not in the clinic. Wearable sensor technologies may provide meaningful objective data for the clinical practice as these findings demonstrate. Technologies offering remote patient monitoring show great promise to play a leading role in the evolving clinical landscape of telemedicine. Early data suggests the use of wearable sensor technologies like the PKG may also contribute to the assessment of known non-motor symptoms associated with PD such as detection of abnormal nocturnal disturbances [16, 17].

Future prospective research will need to address the impact this technology will have on clinical outcomes, resource management, and access to specialized care if such devices are to be widely used.

Additionally, while we found patient engagement in the use of wearable devices to be positive, PD treatment is based on a highly individualized and shared decision between the physician and patient; thus, future research should also incorporate patient perspectives on the use of wearable technology and the quality of care they receive.

Wearable technology continues to evolve. The current version of the PKG System now offers additional quantitative information that was not available during our evaluation, which may provide additional clinical decision support for the clinical management of PwP. Percent Time Immobile (PTI) is a summary score for immobility validated by Kotschet, et al. who studied daytime sleepiness, a common symptom in PwP. They used the PKG to detect 2-minute periods of immobility, which had a 85.2% concordance with the detection of sleep by ambulatory daytime polysomnography, (*p* < 0.0001 Chi Squared). High Epworth Sleepiness Scores (ESS) were associated with the PTI score (*p* < 0.01 Mann Whitney U) during the day (between 0900 and 1800 h). The authors demonstrate immobility is a surrogate marker of daytime sleep in PD, confirmed by correlation with known, standard sleep assessments [18]. Percent Time in Tremor (PTT) is a summary score for the ambulatory assessment of tremor validated by Braybrook, et al. who demonstrated the sensitivity and selectivity of a PTT summary score of≥0.8% was 92.5% and 92.9% respectively in identifying tremor. The advantage of continuous recording of tremor in relationship to a record of bradykinesia and medication consumption means the emergence of tremor can be more readily linked to “off” periods and the threshold of bradykinesia associated with emergence of tremor can aid in the quantification of tremor dominance. Furthermore, it aids in untangling the confusion some patients have between tremor and dyskinesia. The authors propose the PKG’s method of representing tremor has the potential to be a practical clinical tool in conjunction with the other scores presented by the PKG in the management of PD [19].

Since the completion of this project, two panels of movement disorder specialists published consensus statements that serve as a basis for objective measurement to be incorporated into clinical practice guidelines. One panel provided a rationale for objective measurement use in treating PwP and a rationale for treating to an objective target along with potential PKG targets [20]. Another panel specifically discussed the role of the PKG in the clinical management of PwP and provided detailed clinical scenarios where the objective data gained from the PKG can support clinical decision-making such as in the care of patients who are poor historians, have difficulty characterizing motor symptoms, have excessive daytime sleepiness, and in the optimization of new therapies. The panellists concluded early clinical evidence and expert opinion suggest there is a role for the PKG in influencing and enhancing clinical decision-making in the care of PwP and anticipate continued adoption in the coming years [21].

### Conclusion

The PKG provided novel, additional information beyond that captured during a routine clinic visit sufficient to change medical management. Physicians in a movement disorders clinic adjusted treatment nearly a third of the time based on the real-time clinical status captured during objective continuous monitoring outside the clinic setting, rather than solely based on the traditionally obtained historical survey provided during a routine clinical visit. The use of the PKG may provide for better informed therapeutic decisions, improving the quality of life for patients with Parkinson’s disease.

## CONFLICT OF INTEREST

The authors declare there are no conflicts of interest relevant to this work.
